# Multi-omics analysis and experiments uncover the function of cancer stemness in ovarian cancer and establish a machine learning-based model for predicting immunotherapy responses

**DOI:** 10.3389/fimmu.2024.1486652

**Published:** 2024-12-11

**Authors:** Zhibing Liu, Lei Han, Xiaoyu Ji, Xiaole Wang, Jinbo Jian, Yujie Zhai, Yingjiang Xu, Feng Wang, Xiuwen Wang, Fangling Ning

**Affiliations:** ^1^ Department of Oncology, Binzhou Medical University Hospital, Binzhou, Shandong, China; ^2^ Department of Oncology, Qilu Hospital of Shandong University, Jinan, Shandong, China; ^3^ Department of Reproductive Medicine, Binzhou Medical University Hospital, Binzhou, Shandong, China; ^4^ Department of Oncology, Huashan Hospital Fudan University, Shanghai, China; ^5^ Department of Interventional Vascular Surgery, Binzhou Medical University Hospital, Binzhou, Shandong, China

**Keywords:** cancer stemness, immunotherapy, ovarian cancer, TME, snrpe

## Abstract

**Background:**

The heterogeneity of cancer makes it challenging to predict its response to immunotherapy, highlighting the need to find reliable biomarkers for assessment. The sophisticated role of cancer stemness in mediating resistance to immune checkpoint inhibitors (ICIs) is still inadequately comprehended.

**Methods:**

Genome-scale CRISPR screening of RNA sequencing data from Project Achilles was utilized to pinpoint crucial genes unique to Ovarian Cancer (OV). Thirteen publicly accessible OV transcriptomic datasets, seven pan-cancer ICI transcriptomic cohorts, and one single-cell RNA dataset from melanoma patients treated with PD-1 were utilized to scale a novel cancer stemness index (CSI). An OV single-cell RNA dataset was amassed and scrutinized to uncover the role of Small Nuclear Ribonucleoprotein Polypeptide E (SNRPE) in the tumor microenvironment (TME). Vitro experiments were performed to validate the function of SNRPE in promoting proliferation and migration of ovarian cancer.

**Results:**

Through the analysis of extensive datasets on ovarian cancer, a specific gene set that impacts the stemness characteristics of tumors has been identified and we unveiled a negative correlation between cancer stemness, and benefits of ICI treatment in single cell ICI cohorts. This identified gene set underpinned the development of the CSI, a groundbreaking tool leveraging advanced machine learning to predict prognosis and immunotherapy responses in ovarian cancer patients. The accuracy of the CSI was further confirmed by applying PD1/PD-L1 ICI transcriptomic cohorts, with a mean AUC exceeding 0.8 for predicting tumor progression and immunotherapy benefits. Remarkably, when compared to existing immunotherapy and prognosis markers, CSI exhibited superior predictive capabilities across various datasets. Interestingly, our research unveiled that the amplification of SNRPE contribute to remodeling the TME and promoting the evasion of malignant cells from immune system recognition and SNRPE can server as a novel biomarker for predicting immunotherapy response.

**Conclusions:**

A strong relationship between cancer stemness and the response to immunotherapy has been identified in our study. This finding provides valuable insights for devising efficient strategies to address immune evasion by targeting the regulation of genes associated with cellular stemness.

## Introduction

Continuous progress in immuno-oncology, including the creation of checkpoint inhibitors and CAR-T cell therapy, provide hopeful approaches for fighting tumors through the stimulation of the body’s natural defense system (1). However, a significant number of patients do not experience the expected benefits from immunotherapy, highlighting the urgent need to identify the the population eligible for immunotherapy at this stage.

The efficacy of immunotherapy relies on a complex network involving multiple modulators, including the tumor immune microenvironment and genetic heterogeneity. Previous research has primarily focused on analyzing these factors through RNA sequencing of intact tumor tissue (2, 3). However, the variability in the tumor immune microenvironment across different cancer types and individuals, as well as the genetic heterogeneity of tumors, present challenges in accurately predicting patient responses to immunotherapy (4). While certain biomarkers like tumor mutation burden (TMB) have been linked to immunotherapy responses, they may not always accurately predict the effectiveness of specific immunotherapy treatments (5). This underscores the importance of developing robust markers and optimizing combinations of biomarkers to better stratify patients for optimal therapeutic outcomes.

Cancer stem cells contribute to the initiation, progression, and spread of tumors (6). Recently, research has shown a strong link between stem cell characteristics and the ability of cancer cells to evade the immune system and resist treatment (7). Previous study demonstrated a negative correlation between cancer stemness and immune cell infiltration in 21 solid cancers and indicated that high level of stemness have a negative impact on the effectiveness of ICI treatment across various cancer types (8–11). Nevertheless, the connection between tumor stemness and ICI response in ovarian cancer has been disregarded.

This study utilized integrative analyses of transcriptome and CRISPR cell line datasets to identify specific cancer stemness-related mRNAs of ovarian cancer. We also found a negative relationship between cancer-intrinsic variability, cancer stemness, and outcomes of ICI treatment in single-cell SKCM ICI cohorts (12). Subsequently, a CSI was developed by analyzing 13 ovarian cancer cohorts with 2407 patients. The accuracy of CSI in predicting immunotherapy response was assessed using 7 independent anti-PD-1/PD-L1 ICI cohorts with 929 patients and the submap algorithm. We observed a significant inverse correlation between CSI and intrinsic variations, including TMB, mutations, copy number variations, and Homologous Recombination Defects (HRD). Furthermore, combining CSI with TMB was found to improve the predictive accuracy of immunotherapeutic efficacy. Of note, a pivotal gene, SNRPE, was identified as having a promoting effect on tumor growth. This finding suggests that SNRPE could be a potential novel target for immunotherapy in the future. Collectively, our detailed analysis offers valuable insights into the role of cancer stemness in immunotherapy for ovarian cancer.

## Methods

### Acquisition and preprocessing of extensive ovarian cancer datasets

The Cancer Genome Atlas (TCGA) dataset on ovarian cancer RNA sequencing and survival data was retrieved from the UCSC Xena database (13). Additionally, 12 GEO cohorts focusing on ovarian cancer (GSE13876, GSE138866, GSE140082, GSE14764, GSE17260, GSE18520, GSE19829, GSE26712, GSE31245, GSE49997, GSE63885, GSE9891) were acquired, each containing detailed survival information.

### Collection of immunotherapy-associated datasets

The research gathered various sets of data from groups of patients treated with anti-PD-L1/PD-1 medications to investigate the correlation between cancer stem cell characteristics and the effectiveness of immunotherapy. The cohorts included the following: Rose TL cohort (14) (GSE176307: ICB treated metastatic urothelial cancer), Jung H cohort (15) (GSE135222: anti-PD-1/PD-L1 treated non-small cell lung carcinoma), Riaz N cohort (16) (GSE91061: anti-CTLA4 and PD-1 treated melanoma), Liu/VanAllen cohort (phs000452.v3: anti-PD1/CTLA4-treated metastatic melanoma) from the dbGaP database, Necchi cohort (17) (IMvigor210: Atezolizumab treated advanced or metastatic urothelial carcinoma) obtained using the “IMvigor210CoreBiologies” R package, Wang GY cohort (anti-PD-1/PD-L1 treated melanoma), and Braun DA cohort (anti-PD-1 treated advanced clear cell renal cell carcinoma). Gene expression and clinical data were also gathered for these immunotherapy-treated datasets. The details of all cohorts used in this study can be found in **Supplementary Table S1**.

### Collection of single cell datasets for OV and ICI-treated SKCM

Gene expression profiles of single cell OV dataset were preprocessed and retrieved from the GEO database with accession number GSE184880 (18). The dataset consisted of five non-malignant tissues and seven high-grade serous ovarian cancer tissues. Moreover, an examination was conducted on a melanoma cohort to explore the correlation between cancer cell stemness and the efficacy of immunotherapy. This cohort comprised data on both ICI response and single-cell RNA sequencing, sourced from GEO under accession number GSE115978 (12).

### Identifying essential genes for OV

The CRISPR screening of OV cells at a genome-wide level was acquired through the DepMap portal (https://depmap.org/portal/download). Utilizing the CERES algorithm, dependency scores were computed for about 17,000 potential genes (19). Genes deemed essential demonstrated a CERES score below -1 in 75% of the OV cell lines (n = 73).

### Development and validation of CSC prediction model

**Figure 1:**
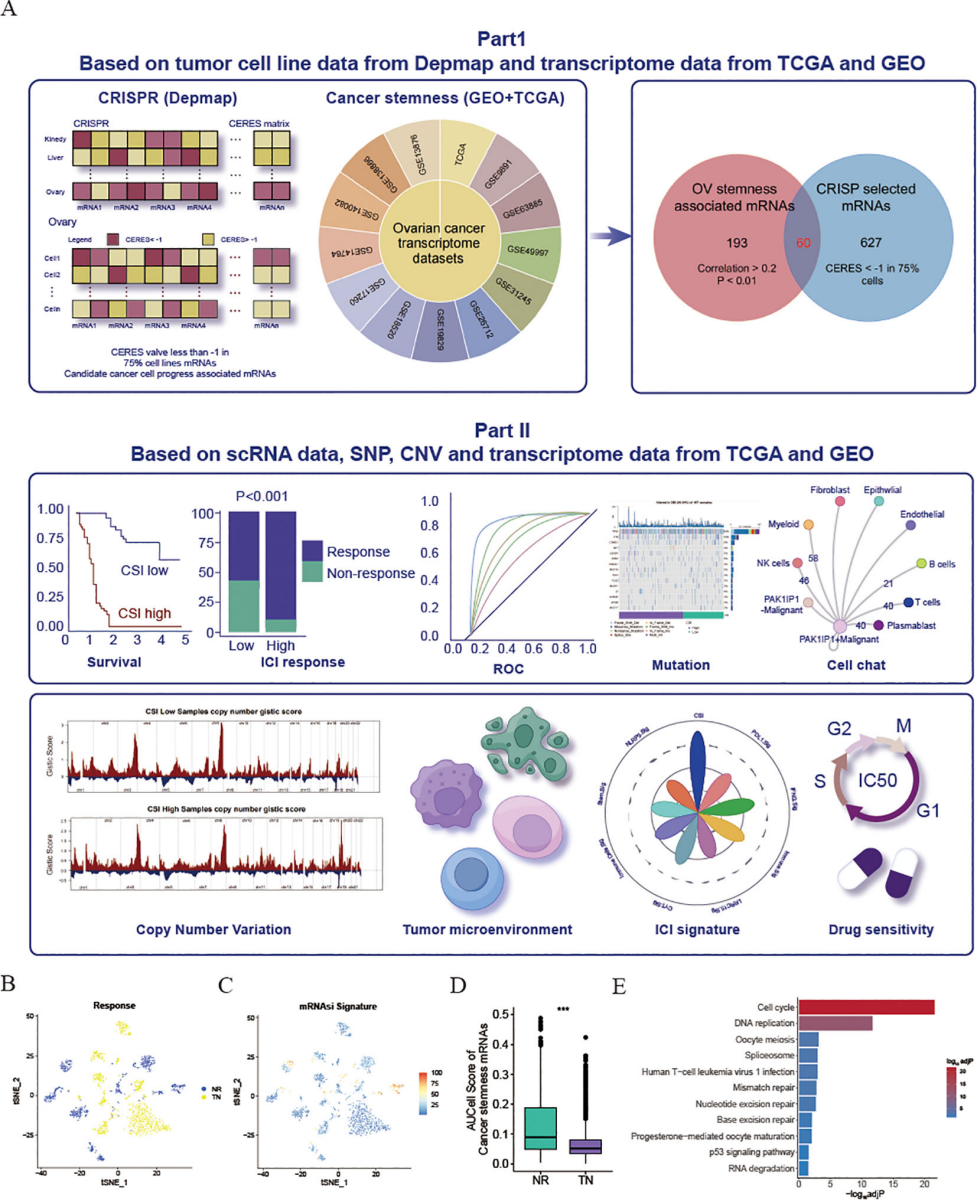
Exploration and validation of an inverse relationship between ovarian cancer stemness and ICI response. **(A)** Schematic representation of the process for identifying mRNA markers associated with cancer’s intrinsic heterogeneity and stemness, and the development of predictive models using various machine learning techniques. **(B–D)** Visualization of t-Distributed Stochastic Neighbor Embedding (tSNE) plots for malignant cells in the SKCM dataset. **(B)** Malignant cells categorized by response phenotype in the tSNE plot. **(C)** tSNE plot depicting the AUCell scores of cancer stem cell (CSC)-related gene sets in malignant cells, with red indicating higher scores (indicative of high stemness) and blue representing lower scores (indicative of low stemness). **(D)** Box plot illustrating the distribution of AUCell scores across response phenotypes (non-responders vs. treatment-naïve patients) in the SKCM cohort. The median values are marked at the center of the box plot, with the box boundaries representing the 25% and 75% quantiles (Wilcoxon test; *** P < 0.001). Abbreviations: NR, non-responders; TN, treatment-naïve patients. **(E)** KEGG enrichment of cancer stemness associated mRNAs.

An innovative pipeline was developed to construct a predictive model for Cancer stem cells (CSC), illustrated in **Figure 1A**. Initially, by utilizing the CERES algorithm with cell line data, we pinpointed 687 mRNAs that displayed an association with the survival and progression of ovarian cancer cells. Subsequently, we computed mRNA stemness indices (10) across 12 GEO datasets, the TCGA-OV dataset and evaluated the relationship between total mRNA and mRNA expression-based stemness index (mRNAsi). We then identified the mRNAs showing significantly positive correlations in at least half of the cohorts (7 out of 13) as mRNAsi-associated mRNAs (Cor>0.2 and P<0.01), resulting in the discovery of 60 mRNAsi-associated mRNAs (**Supplementary Tables S2, S3**).

Finally, 8 ovarian cancer (OV) cohorts were used in the creation of a predictive model for CSCs. To accomplish this, a variety of machine learning techniques were employed, including random forest (RSF), elastic net (Enet), gradient boosting (GBM), ridge regression, Stepcox, plsRcox, CoxBoost, and SuperPC.

### Prediction of immunotherapy outcomes using TIDE webserver

To evaluate the effectiveness of PD-1/CTLA4 immunotherapy, our first step involved the calculation of scores for tumor immune dysfunction and exclusion (TIDE). This analysis was performed using the adjusted expression data collected from patients with ovarian cancer. The resultant matrix of expression profiles was then submitted to the TIDE database website (http://tide.dfci.harvard.edu/) to assess the response of the patients (20). Next, we employed the submap algorithm available on the GenePattern website to determine the differences in response likelihood between the low- and high-CSI groups.

### Identification of optimal therapeutics for low and high CSI groups and drug sensitivity analysis

By analyzing gene expression profiles, drug sensitivity prediction in cell lines was achieved using the ‘oncoPredict’ R package and the calcPhenotype method. To estimate drug IC50, a ridge regression model was developed utilizing gene expression profiles of cell lines acquired from GDSC through the pRRophetic algorithm (21).

### Cell lines

Human ovarian cancer cell lines, specifically OVCAR-3, A2780, and SK-OV-3, were supplied by the Cell Bank of the Committee for Conservation of Typical Cultures, which is part of the Chinese Academy of Sciences. These cell lines were cultured using Dulbecco’s Modified Eagle Medium (DMEM) from Gibco (New York, USA) and enriched with 10% fetal bovine serum. Furthermore, the culture medium was supplemented with 100 IU/mL penicillin and streptomycin, both of which were also procured from Gibco (New York, USA).

### IHC

Following the removal of paraffin, the sections embedded in paraffin were subjected to a treatment with 3% hydrogen peroxide at 26°C for 10 minutes to suppress the activity of endogenous peroxidase. Next, the sections were blocked using 10% goat serum to avoid non-specific binding. Afterward, the sections were incubated overnight at 4°C with primary antibodies. Subsequently, rabbit secondary antibodies were applied to the sections, which were then stained using DAB.

### Knockout and overexpression in ATC cell lines

Lentiviral vectors designed for the overexpression of SNRPE were sourced from Genechem in Shanghai, China. Stable transfection of cells with these SNRPE-overexpressing lentiviruses, along with the corresponding control plasmids, was performed to induce puromycin resistance. Following the instructions provided by the manufacturer, selection of stable transfectants was carried out using 2 μg/mL puromycin over a period of 7 days to establish stable SNRPE-overexpressing cell lines. Biotend Co., Ltd. synthesized the siRNA targeting SNRPE. The siRNA, at a concentration of 50 nM, was transfected into cells using the Lipofectamine 3000 transfection kit provided by Thermo Fisher Scientific in Waltham, Massachusetts, USA, with a 24-hour incubation period.

### Western blotting

To conduct western blot analysis, cells in culture were washed with ice-cold PBS before extracting total cell protein lysates at 4°C with RIPA lysis buffer (Beyotime, Shanghai, China) supplemented with 1% protease inhibitor cocktail (MedChemExpress, New Jersey, USA). Following centrifugation at 12,000 g for 20 minutes at 4°C, the supernatant containing proteins was gathered and mixed with loading buffer. The samples were then subjected to separation by 10% SDS-PAGE and transfer onto a PVDF membrane. The membrane was then blocked for 2 hours at room temperature with 5% skim milk before incubating overnight at 4°C with primary antibodies. After rinsing with Tris Buffered Saline, the membrane was exposed to secondary antibodies for detection of protein bands using enhanced chemiluminescence reagents (Beyotime, Shanghai, China). Antibodies used in the analysis included SNRPE (20407-1-AP, Proteintech, Wuhan, China) and GAPDH (60004-1-Ig, Proteintech, Wuhan, China).

### Assessment of cell proliferation, colony formation, and migration abilities

To evaluate cell proliferation, 2×10^3 cells were introduced into each well of a 96-well plate and maintained for the required period. Afterward, each well was treated with 10 μl of CCK-8 reagent (Dojindo Molecular Technologies, Kumamoto, Japan) and left to incubate for one hour. The absorbance was then recorded at a wavelength of 450 nm (OD450) for further analysis.

To assess the ability of colonies to form, a range of 500 to 2000 cells were placed in each well of a 6-well plate and left to incubate for around one week. Upon detection of colonies with over 50 cells, they were treated with 0.2% crystal violet for a duration of 30 minutes. Following three rounds of washing with PBS, the colonies were both captured in pictures and tallied for measurement.

To evaluate the migratory potential of cells, a total of 40,000 cells were suspended in 200 μL of culture medium and placed in the upper compartment of Transwell plates from BD Biosciences. At the same time, 600 μL of culture medium with 10% FBS was introduced into the lower compartment. After an overnight incubation at 37°C, the cells located beyond the Transwell membrane were fixed using 4% paraformaldehyde for half an hour and subsequently stained with 0.25% crystal violet for an additional 30-minute period. Following the removal of cells from the interior of the chamber, the migrated cells outside the membrane were visualized and quantified.

### Statistical analysis

We utilized the Wilcoxon test to assess various attributes of the high- SNRPE and low- SNRPE groups. The Chisq test was employed to scrutinize the variability in immunotherapy response among the high-CSI and low-CSI groups. The correlation between mRNA and mRNAsi was investigated through the calculation of Pearson’s correlation coefficient. Kaplan-Meier survival analysis was performed to explore the connection between CSI, SNRPE, and survival, utilizing the log-rank test to determine the significance of observed distinctions. To assess the prognostic and immunotherapy advantages of CSI, time-dependent receiver operating characteristic (ROC) curves were generated with the assistance of the ‘pROC’ R package (22) being utilized. Key factors influencing immunotherapy efficacy were identified using xGboost, a scalable tree boosting system. Patients were grouped based on the optimal threshold established by the ‘survminer’ R package. Statistical significance was defined by a significance level of P or adjP < 0.05.

## Results

### Revelation of the link between cancer stemness and immunotherapy resistance through scRNA ICI cohort

Considering the potential influence of cancer stemness on the resistance to ICIs, a comprehensive analysis was conducted on 13 transcriptome datasets related to OV obtained from the GEO and TCGA databases. The mRNAsi was calculated for each patient (10). By utilizing the Pearson correlation coefficient, mRNAs that exhibited a significant relationship with mRNAsi across multiple samples (Cor>0.2 & P<0.01) were identified. Subsequently, 253 mRNAs that detected in more than 50% of the datasets (7 out of 13) were considered as tumor stemness-associated mRNAs. Moreover, to pinpoint crucial candidate genes involved in OV malignancy, an in-depth examination of CRISPR-based loss-of-function screens was undertaken on a global scale based on DepMap database. As a result, a total of 687 genes essential for the survival of 73 OV cell lines (CERE score < -1 in 75% OV malignant cells) were identified. Of these, 60 mRNAs were selected through the overlap of OV mRNAsi-associated mRNAs with the mRNAs highlighted by CRISP (**Supplementary Table S3**). All these genes associated with cancer stemness were chosen for further study. To validate the influence of cancer stemness associated genes on immunotherapy effectiveness, a previously published scRNA-seq dataset of PD1 ICI-treated patients with melanoma (SKCM) was initially employed to investigate the correlation between cancer stemness and ICI responses. After excluding individuals lacking data on malignant cells, a total of 23 patients from this cohort were included, comprising 10 non-responders (NR) and 13 treatment-naïve (TN) patients. Ideally, a comparison of cancer stemness between responders (R) and NR to ICI treatment would have been preferred. However, the dataset available did not contain specific data on responders. Given that treatment-naïve patients may consist of both potential responders and non-responders, our next step was to compare the stemness levels between the NR group and the TN group, as previously described. As depicted in **Figures 1B, C**, the NR subcategory displayed a higher frequency of cancer cells with increased stemness rankings. Further investigation revealed that individuals from the NR subgroup had significantly higher levels of stemness (P < 0.001, **Figure 1D**), indicating an inverse relationship between cancer intrinsic driver and stemness with immune checkpoint inhibitor outcomes. Furthermore, we also found that these cancer stemness related genes significantly enriched Cell proliferation-related pathways, including Cell cycle and DNA replication (**Figure 1E**), indicating that tumor stemness-related genes may stimulate tumor cell proliferation.

### Establishing the cancer stemness index through machine learning methodologies

**Figure 2:**
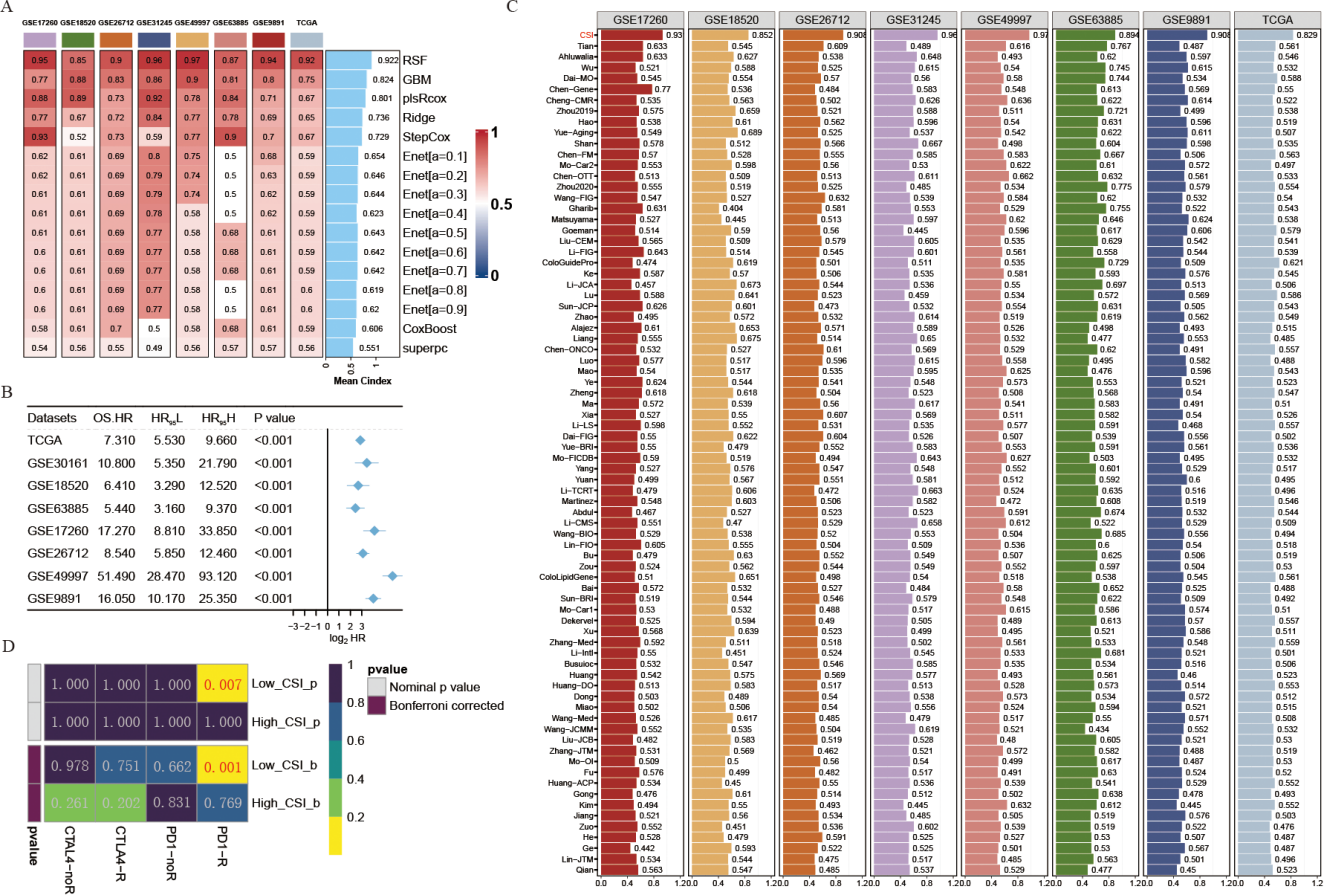
Development of a cancer stemness index utilizing extensive scRNA-seq and bulk RNA-seq datasets. **(A)** C-index of eight algorithms across eight validation cohorts. **(B)** Univariate Cox regression analysis of the RSF score in eight OV cohorts. **(C)** Estimated response rates to immune checkpoint inhibitors (PD-1/PD-L1) across different CSI groups (R: Response, NR: No Response). **(D)** ROC values comparing the predictive accuracy of the CSI and 79 other models for clinical status in eight OV cohorts.

To further develop a prediction model for CSI, nine machine learning algorithms were used with a combination of six GEO OV datasets and the OV TCGA dataset. Subsequently, we calculated the risk score for each sample in the eight cohorts, which included survival data, using these predictors. The performance was evaluated by determining the average C-index for each algorithm. Interestingly, most of these predictors showed a considerably high average C-index (**Figure 2A**). This finding can be partly attributed to the exceptional quality of our cancer stemness markers. Among all the models, random forest (RSF) demonstrated the highest level of precision (average C-index = 0.922, **Figure 2A**) and was chosen as the definitive CSI. Additionally, through univariate cox analysis, a significant correlation between high CSI in the seven cohorts and poor survival outcomes was established (P<0.05, **Figure 2B**).

The progress in next-generation sequencing and large-scale data mining technologies has facilitated the thorough investigation and advancement of gene expression-derived markers that are able to forecast prognosis results. To thoroughly assess the effectiveness of the CSI in comparison to alternative markers, we methodically compiled previously published markers from the past decade. A total of 79 markers were analyzed in this research (**Supplementary Table S4**). Notably, the reliability of the CSI in predicting survival outcomes exceeded that of all other models across eight different OV cohorts, achieving an average AUC > 0.9 in the mentioned cohorts (**Figure 2C**).

Given the importance of cancer stemness mRNAs in predicting the success of tumor immunotherapy, we utilized the submap algorithm available on GenePattern website to predict the probability of immune therapy response based on high- and low- CSI groups. A noteworthy finding was revealed when individuals from the low CSI group exhibited a significant reaction to PD-1 immunotherapy (P = 0.007, Bonferroni corrected P < 0.001, **Figure 2D**), demonstrating the remarkable predictive power of the CSI model in the context of PD-1 immunotherapy efficacy.

Collectively, these results suggest that CSI could function as predictive markers for the prognosis of ovarian cancer, with higher robust than other models. In addition, CSI can also be used as a prognostic indicator of PD1 immunotherapy response.

### Cancer stemness index demonstrates predictive capabilities for immunotherapy outcomes

**Figure 3:**
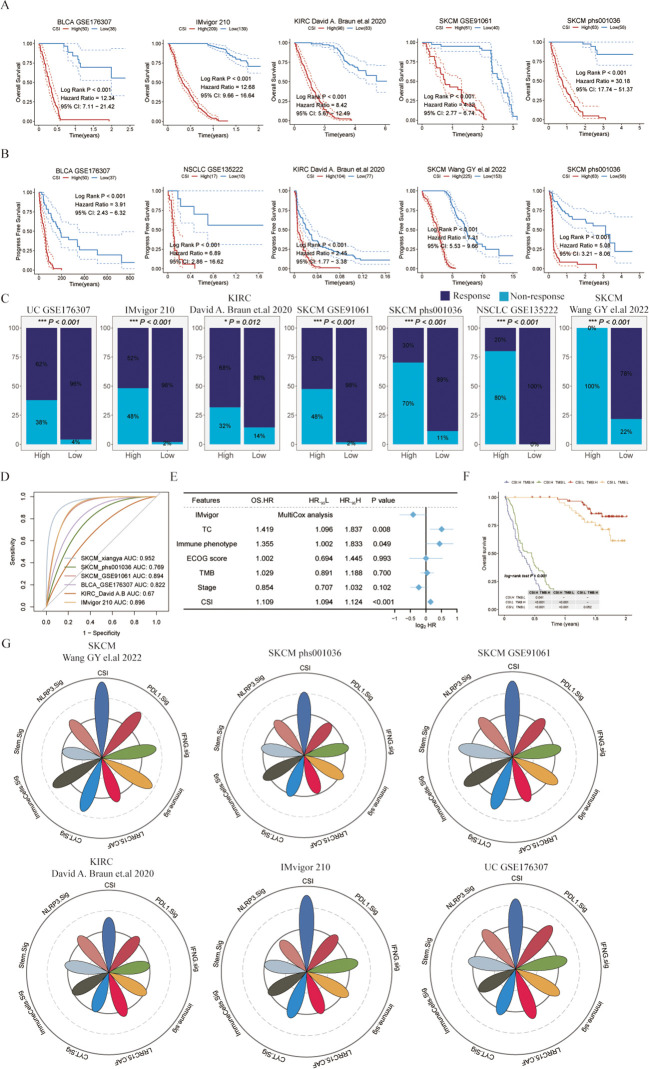
Evaluating the cancer stemness index as a potent prognostic tool for immunotherapy outcomes across various cancer types. **(A)** Kaplan-Meier survival curves depicting overall survival of patients undergoing immunotherapy in bladder cancer (UC, GSE176307), metastatic urothelial carcinoma (IMvigor210), kidney renal clear cell carcinoma (KIRC, David **(A)** Braun et al., (26)), and melanoma (SKCM, GSE91061 and phs000452.v3.p1). **(B)** Kaplan-Meier curves for progression-free survival of patients receiving immunotherapy in bladder cancer (UC, GSE176307), non-small cell lung cancer (NSCLC, GSE135222), KIRC (David **(A)** Braun et al.), and melanoma (SKCM, Wang GY et al., 2022 and phs000452.v3.p1). **(C)** Immunotherapy response rates in patients categorized by different CSI groups, with response defined as complete response (CR) or partial response (PR), and non-response as progressive disease (PD) or stable disease (SD). **(D)** ROC curves of the CSI for predicting response status in various immune checkpoint inhibitor (ICI) cohorts. **(E)** Multivariate Cox regression analysis of the CSI and clinical features in metastatic urothelial carcinoma (IMvigor210). **(F)** Kaplan-Meier survival curves for overall survival in different patient groups within the IMvigor210, with log-rank P values comparing each pair of groups displayed in the table. **(G)** Radar plot comparing the ROC values of eight ICI response prediction models and the CSI.

To further verify the predictive performance of CSI on the therapeutic effect of PD-1 ICIs, we collected different datasets associated with PD-1/PD-L1 immune checkpoint inhibitors. Our results consistently indicated that patients diagnosed with specific cancers (such as SKCM, UC, KIRC, or metastatic urothelial carcinoma) who had lower CSI scores experienced notably enhanced overall survival (OS) or progression-free survival (PFS) following immunotherapy compared to those with higher CSI scores (**Figures 3A, B**). This suggests that higher CSI scores may impede the benefits of PD-1 immunotherapy. Furthermore, the response to PD-1/PD-L1 ICI therapy varied between patients with high and low CSI scores. Individuals with higher CSI scores exhibited suboptimal response to the treatment, whereas over half of those with lower CSI scores responded positively (**Figure 3C**). More specifically, the group with higher CSI scores predominantly displayed no response (progressive disease or stable disease), whereas the group with lower CSI scores mainly demonstrated a response (complete response or partial response). Significantly, our analysis indicated that CSI serves as a reliable predictor of PD-1/PD-L1 ICI immunotherapy response, as demonstrated by the area under the curve (AUC) values. The AUC curve portrayed outstanding predictive performance, with an average AUC > 0.8 across the six cohorts examined (**Figure 3D**). Additionally, we performed further analysis using the IMvigor210 dataset and observed that even upon excluding samples with incomplete clinical data, CSI remained a robust predictor of immunotherapy outcomes. Intriguingly, it held greater significance compared to parameters such as PD-L1 expression in tumor cells (TC), immune phenotype, ECOG score, Stage, or tumor mutation burden (TMB), as indicated by a multivariate Cox regression analysis (refer to **Figure 3E**). To extend the clinical utility of our model, we explored the potential benefits of combining CSI with other commonly utilized markers of immunotherapy response. Specifically, we investigated the synergistic effects of CSI and TMB, a well-known indicator of immunotherapy effectiveness. Our findings revealed that patients exhibiting low CSI scores and high TMB levels experienced the most favorable outcomes with immunotherapy treatment, whereas those with elevated CSI scores demonstrated the least benefits from such therapies (**Figure 3F**). Additionally, we conducted a comparative analysis of CSI with established signatures for predicting immunotherapy response. Notably, CSI outperformed various signatures, including IFNG.Sig (23), Immune.Sig (23), ImmuneCells.Sig (16), PDL1.Sig (24), LRRC15.CAF.Sig (25), NLRP3.Sig (26), Stem.Sig (11), and CYT.Sig (27) in six PD1/PD-L1 immunotherapy cohorts, while the majority of these signatures exhibited optimal performance in only one or two cohorts (**Figure 3G**).

In conclusion, our research offers important insights into the predictive significance of CSI on immunotherapy results. Elevated CSI levels could potentially hinder the advantages of immunotherapy, whereas lower CSI levels have been linked to better survival rates and treatment responses. Integrating CSI with TMB could potentially improve the classification of patients for immunotherapy.

### Intrinsic somatic mutations and copy number variation patterns of different CSI group patients

Examining somatic mutations and copy number variations (CNVs) as factors influencing both antitumor immunity and tumor advancement (28), we analyzed the most commonly mutated genes in various CSI categories (see **Supplementary Figure S1A**). Among OV patients, TP53 exhibits the greatest mutation rate, trailed by TTN and CSMD3. TMB stands for the tally of somatic non-synonymous mutations in a specific genetic area, usually expressed as mutations per megabase (mut/Mb). Prior research has demonstrated a negative correlation between TMB and the efficacy of immunotherapy (29). Notably, our investigation revealed that the TMB levels were greater in patients from the low-CSI category compared to those in the high-CSI group (**Supplementary Figure S1B**, P<0.05). Consistent with this, the SNV neoantigens and rate of nonsilent mutations were notably elevated in the low-CSI group compared to the high-CSI cohort. (**Supplementary Figures S1C, D**, P<0.05). In addition, Homologous Recombination Repair (HRR), a key mechanism for repairing DNA double strand breaks in cells, plays a critical role in maintaining the stability and integrity of the genome. HRD refers to conditions that occur when this repair mechanism is impaired, which may be due to genetic mutations in key repair proteins (such as BRCA1 and BRCA2) or dysfunction of other regulators. Prior research has indicated that targeting HRD defects can be an effective strategy for combating cancer. This includes not only conventional treatments like chemotherapy and radiotherapy, which cause DNA damage, but also newer approaches such as targeted therapies and immunotherapies (30, 31). Consistently, our research revealed that the Homologous Recombination Defects rating was notably elevated in the low CSI group compared to the high CSI group (**Supplementary Figure S1E**, P <0.001). Furthermore, to assess the prevalence of CNV across various CSI groups, we subsequently utilized the Genomic Identification of Significant Targets in Cancer (GISTIC) algorithm. Notably, we observed that the amplification GISTIC score was greater in patients from the low CSI group than in those from the high CSI group (**Supplementary Figure S1F**).

Collectively, these findings further elucidate the rationale behind the improved immunotherapeutic efficacy observed in patients with lower tumor stemness and demonstrate that CSI could serve as a prognostic indicator for predicting the therapeutic benefits of ICIs in ovarian cancer.

### The amplification of SNRPE promotes the progress of ovarian cancer

**Figure 4:**
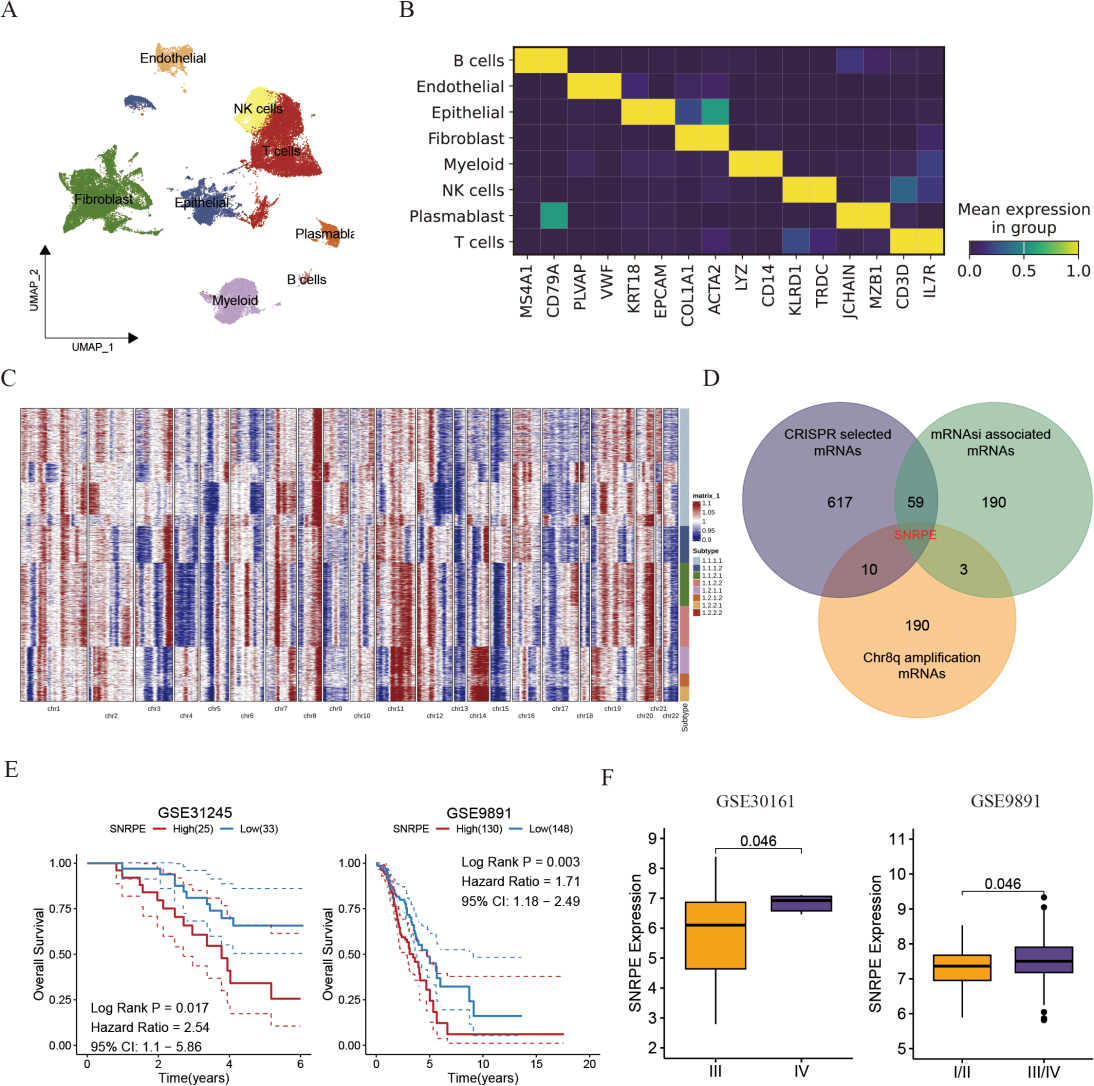
Single cell analysis uncovered the amplification of SNRPE promotes the progress of ovarian cancer. **(A)** UMAP plot showing the composition of 8 main subtypes derived from OV malignant cells. **(B)** Heatmap showing expression of each cell markers in each cell type. **(C)** Heatmap showing copy number variation of reference cells and epithelial cells. **(D)** Venn diagram showing the overlap between intratumoral heterogeneity (8q amplification) driven genes, cancer cell develop associated genes and cancer stemness associated genes. **(E)** Kaplan-Meier curves for overall survival of ovarian patients between SNRPE -high and -low groups (GSE31245 and GSE9891). **(F)** Box plots comparing SNRPE expression between early and advanced OV groups.

To explore the clonal architecture and cell origins of ovarian malignant cells, we initially obtained a single-cell RNA profile from ovarian carcinoma. After filtering cells with a minimum expression of 200 genes and excluded those with over 20% expression of mitochondrial genes, we grouped the residual cells into eight major cell types based on traditional biomarkers. As described in **Figures 4A, B** all cells divided into diverse cell populations including B cells (MS4A1, and CD79A), Endothelial cells (PLVAP, and VWF), Epithelial cells (KRT18, and EPCAM), Fibroblasts (COL1A1, and ACTA2), Myeloid cells (LYZ, and CD14), NK cells (KLRD1, and TRDC), Plasmablast cells (JCHAIN, and MZB1), as well as T cells (CD3D, and IL7R).

Subsequently, the inferCNV algorithm was utilized to assess copy number variations (CNV) and clonality in ovarian malignant cells derived from epithelial cells (ECs). Among the 2807 ECs from THCA tissues, 6230 displayed high CNV scores, indicating malignancy (**Figure 4C**). Notably, amplifications in chromosomal 8q were identified as specific driving variations in ovarian cancer, with SNRPE amplification in chromosomal 8q being linked to cancer stemness and essential for the survival of ovarian cell (**Figure 4D**). Prior studies have indicated SNRPE’s role in promoting cell growth and advancing high-grade prostate cancer by controlling the expression of the androgen receptor [22740892]. Consistently, patients exhibiting high SNRPE expression consistently demonstrated poorer prognoses compared to those with low SNRPE expression (**Figure 4E**). Additionally, we observed that elevated SNRPE expression was specifically linked to tumor stage progression (**Figure 4F**).

**Figure 5:**
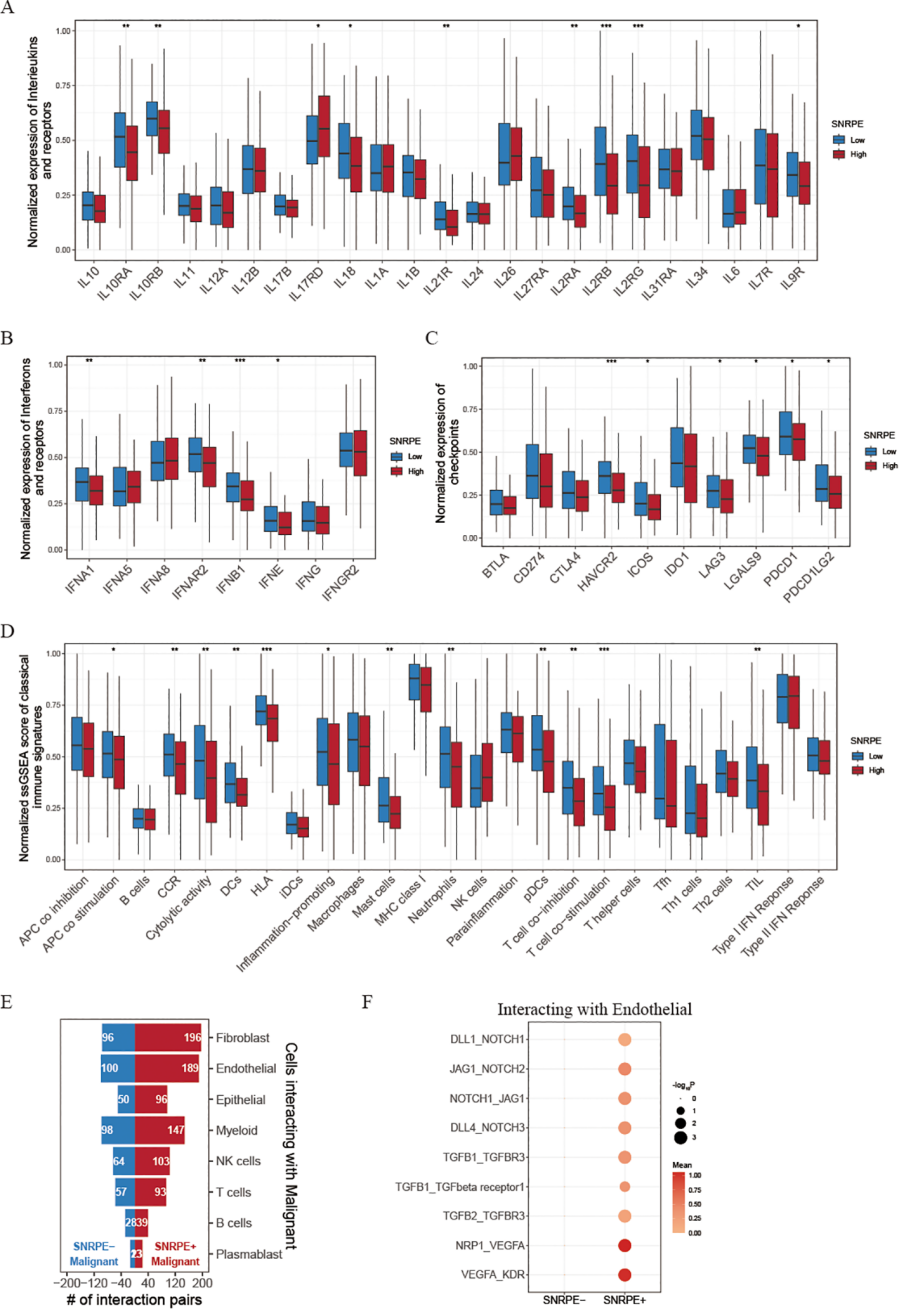
Investigating immune landscapes related to SNRPE expression. **(A, B)** Box plots comparing expression of interleukins, interferons and their receptors between low and high SNRPE groups. **(C)** Box plots for checkpoint expression comparison. **(D)** Normalized ssGSEA scores of classical immune signatures in the two groups. **(E)** Bar plots showing cell communication numbers between SNRPE- or SNRPE+ malignant cells and other cell types. **(F)** Dot plot depicting cell communication pairs of malignant cells (stratified by SNRPE status) with endothelial cells. Wilcoxon test; *P<0.05, **P<0.01, ***P<0.001.

Given that tumor heterogeneity and stemness play a crucial role in immune evasion and response to immunotherapy, we analyzed the TME among high and low SNRPE patients categorized based on the optimal threshold established by the ‘survminer’ R package. Tertiary lymphoid structures (TLS), which serve as germinal centers for immune cells, were analyzed for the expression of various interleukins associated with the formation of TLS and the activation of immune responses. Our findings indicate that the majority of these interleukins exhibited elevated expression levels in the group with high SNRPE. Specifically, we found that patients with low SNRPE expression showcased increased expression of IL10RA, IL10RB, IL18, IL21R, IL2RA, IL2RB, IL2RG and IL9R (**Figure 5A**). Additionally, numerous interferons along with their receptors (for example, IFNE, IFNG, IFNAR2, IFNGR2) as well as the majority of interleukins and their corresponding receptors were linked to immune-activating transcripts. Our discovery revealed that the levels of these interferons and receptors were elevated in the low SNRPE group, a pattern that aligns with the inverse relationship of interleukins within the tumor microenvironment (**Figure 5B**). Furthermore, recognizing the importance of immune checkpoint presence as a critical element in immunotherapy with ICIs, we carried out an additional investigation into the levels of immune checkpoints within two distinct groups. It is worth mentioning that the expression levels of several checkpoints (such as HAVCR2/TIM-3, ICOS, LAG3, LGALS9, PDCD1/PD-1, and PDCD1LG2/PD-L2) were significantly higher in the low SNRPE group compared to the high SNRPE group, indicating that higher expression SNRPE patients may benefit from immunotherapy benefit (**Figure 5C**). We also analyzed classical immune signatures in each sample, and we found that the most immune signatures were lower in high SNRPE group, suggesting that these immune cell and immune function were suppressed (**Figure 5D**). These results indicated that SNRPE might impact the effectiveness of immunotherapy by regulating the expression of immune checkpoints and immune microenvironment factors.

To further explore the mechanism of SNRPE on ovarian malignant cells, we then divided malignant tumor cells into SNRPE+ malignant cells and SNRPE- malignant cells according to whether they expressed SNRPE. Through cell-cell interaction analysis, after eliminating common cell communication pairs, we found that SNRPE+ malignant cells had significantly higher specific cell communication with endothelial cells and fibroblasts than SNRPE-malignant cells (**Figure 5E**). Hence, we speculated that SNRPE+ malignant may promote tumor invasion and migration by promoting endothelial mesenchymal transformation. Interestingly, we found that NOTCH signaling pathway (DLL1_NOTCH1, JAG1_NOTCH2, NOTCH1_JAG1, and DLL4_NOTCH3), TGFβ1 signaling pathway (TGFB1_TGFBR3, TGFB1_TGFbeta receptor1, and TGFB2_TGFBR3) and VEGF signaling pathway (NRP1_VEGFA, and VEGFA_KDR) were significantly activated in cell communication pairs between SNRPE+ malignant cells and endothelial cells (**Figure 5F**). Then, we reclustered the tumor cells and found high expression of SNRPE in the C3 and C4 subpopulations (**Supplementary Figures S2A, B**). Using the HALLMARK pathway scoring, we identified that C3 can be defined as the EMT subpopulation, characterized by high activity in EMT signaling pathways. On the other hand, C4 can be defined as the proliferative subpopulation, characterized by the activation of proliferation-related pathways, including E2F_TARGETS, G2M_CHECKPOINT, MYC_TARGETS_V1, and MYC_TARGETS_V2 (**Supplementary Figure S2C**). Additionally, through analysis of cell-cell communications, we further discovered that the interaction intensity between SNRPE+ malignant cells and endothelial cells was significantly higher than that between SNRPE- malignant cells and endothelial cells (**Supplementary Figure S2D**). These findings further demonstrate how SNRPE enhances tumor cell proliferation and invasion. Furthermore, a univariate Cox regression analysis of the pan-cancer cohorts revealed that SNRPE expression was negatively correlated with prognosis across multiple cancer types, including Adrenocortical Carcinoma (ACC), Head and Neck squamous cell carcinoma (HNSC), Kidney Chromophobe (KICH), Kidney renal clear cell carcinoma (KIRC), liver hepatocellular carcinoma (LIHC), Brain Lower Grade Glioma (LGG), Lung adenocarcinoma (LUAD), and Pheochromocytoma and Paraganglioma (PCPG) (**Supplementary Figure S3A**).

**Figure 6:**
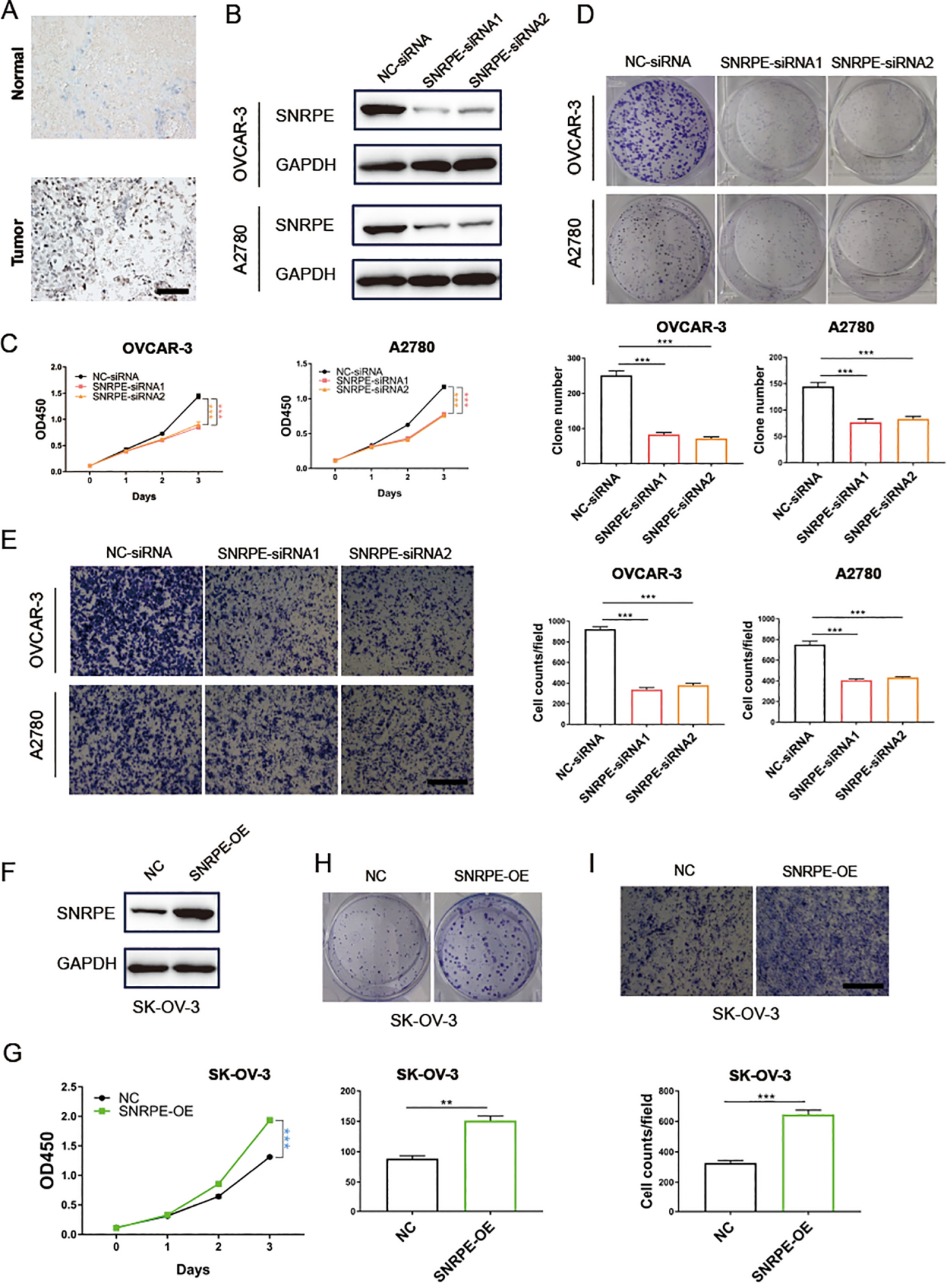
SNRPE promotes proliferation and migration of ovarian cancer *in vitro*. **(A)** Immunohistochemistry analysis revealed a marked increase in CSE1L expression in tumor tissues relative to the adjacent non-cancerous tissues. **(B)** Knockout of SNRPE in OVCAR-3 and A2780 cell lines validated by Western Blotting analysis. **(C)** The proliferative abilities of SNRPE knockout ovarian cancer cells detected with CCK8 assay. **(D)** The clone formation abilities of SNRPE knockout OVCAR-3 and A2780 cell lines. **(E)** The migrating abilities of SNRPE knockout OVCAR-3 and A2780 cell lines evaluated with transwell assay. Scale bar, 100 μm. **(F)** Overexpression of SNRPE validated by Western Blotting analysis in SK-OV-3 cells. **(G)** The proliferative abilities of SNRPE overexpressing SK-OV-3 cells detected with CCK8 assay. **(H)** The clone formation abilities assessed in SK-OV-3 cells upon SNRPE overexpression. **(I)** The migrating abilities of SK-OV-3 cells detected by transwell assay upon SNRPE overexpression. Scale bar, 100 μm. T test; **P < 0.01; ***P < 0.001.

Immunohistochemistry analysis revealed a marked increase in CSE1L expression in tumor tissues relative to the adjacent non-cancerous tissues (**Figure 6A**). To further validate SNRPE’s oncogenic role in ovarian cancer, SNRPE was knocked down in OVCAR-3 and A2780 cell lines, effectiveness confirmed at the protein-level through western blot analyses (**Figure 6B**). Significantly, SNRPE knockdown markedly suppressed cell proliferation in both OVCAR-3 and A2780 cell lines (**Figure 6C**), underscoring SNRPE’s contribution to promoting ovarian cancer cell growth. This was further supported by reduced clonogenic capacity in SNRPE knockdown cells compared to controls (**Figure 6D**), highlighting SNRPE’s involvement in fostering growth in ovarian cancer cells. Notably, transwell migration assays revealed decreased cell migration upon SNRPE depletion in OVCAR-3 and A2780 cell lines (**Figure 6E**).

Concurrently, SNRPE overexpression in SK-OV-3 cells was validated through western blot analyses (**Figure 6F**), demonstrating its significant enhancement of proliferation (**Figure 6G**). Furthermore, SNRPE overexpression notably boosted clonogenic potential and significantly increased migration capability in SK-OV-3 cells (**Figures 6H, I**).

In summary, the amplification of SNRPE can drive the progression of ovarian malignant cells and it may serve as an efficient biomarker in foreseeing the prognosis and immunotherapy response in ovarian cancer.

## Discussion

Tumor heterogeneity and stemness play key roles in influencing tumor immune evasion and the efficacy of immunotherapy. Numerous studies have explored the association between cancer stemness and the ICI response against tumors (6, 7). However, currently, there is no direct evidence linking tumor stemness to the response to ICI in OV. Furthermore, previous research has failed to acknowledge the predictive ability of tumor stemness in determining the response to ICI in OV (11).

In this research, we initially used a stemness index from a previous publication to identify mRNAs linked to tumor stemness by analyzing various omics data, such as transcriptome and CRISPR cell line data (10). Subsequently, to investigate the correlation between tumor stemness and immunotherapy, we examined a single-cell dataset of PD1/PD-L1 ICI-treated cells (12). It should be emphasized that we observed an inverse relationship between cancer stemness and the responses to ICI. CSCs are specialized cells that play a critical role in tumor initiation, progression, and spreading (6). Additionally, our KEGG enrichment analysis of genes associated with cancer stemness showed a significant enrichment in pathways such as Cell cycle, DNA replication, Mismatch repair, Nucleotide excision repair, and Base excision repair. A previous investigation highlighted that the abnormal activation of the cell cycle pathway can lead to an increase in the expression of transcription factors like CDK, MKI67, and p53, which may support the occurrence and sustenance of tumor stemness (32). Besides, improved DNA repair ability readied cancerous cells for harsh surroundings (33). Our findings aligned with prior research and proposed that the gene set linked with cancer stemness which we revealed could strongly and uniquely correspond with cancer stemness.

Then, to assess the impact of tumor stemness genes on the prognosis of ovarian cancer patients, we employed various machine learning techniques to create a predictive model for CSI. This model’s performance was then validated in eight separate datasets using a variety of assessment measures. Ultimately, the RSF model was chosen as the optimal CSI due to its increased stability and accuracy compared to the 79 previously established models. Notably, our research highlighted the CSI’s effectiveness in predicting the response to PD-1 immune checkpoint inhibitors in ovarian cancer (11). Prior investigations have indicated a correlation between tumor stem cells and immune checkpoint inhibitor effectiveness. Building on these findings, we hypothesized that the CSI could be widely applicable for forecasting immunotherapy responses in various cancer types. As a result, we conducted an extensive analysis to evaluate the CSI’s precision in predicting immunotherapy responses in other cancer types. Impressively, the CSI displayed exceptional accuracy in predicting ICI responses across diverse datasets utilizing bulk RNA-Seq data, with an average AUC exceeding 0.8. Additionally, our CSI demonstrated superior predictive capabilities compared to eight existing ICI response prognostic models. Notably, leveraging the IMvigor 210 dataset, we found that the CSI had better prognostic accuracy for post-immunotherapy patients compared to TMB. Our analysis also identified significantly different survival rates between low and high TMB patients. These results highlight the CSI’s impressive predictive ability for both prognosis and immunotherapy outcomes in cases of ovarian cancer.

The quantity of neoantigens on tumor cells is determined by intrinsic variations within the tumor, which in turn impacts the immune system’s ability to recognize and combat the tumor (34, 35). TMB serves as a crucial biomarker for predicting the effectiveness of immune checkpoint inhibitors. Clinical research has consistently shown that patients with high TMB tumors have a higher rate of clinical benefit when treated with these inhibitors (34, 36). Our research revealed a significant negative correlation between TMB levels, SNV neoantigens, nonsilent mutation rates, and CSI. Previous studies have indicated that HRD defects can be targeted by various anti-cancer treatments, including chemotherapy, radiotherapy, targeted therapies, and immunotherapies (30, 31). Notably, the level of HRD was found to be higher in the low CSI group as opposed to the high CSI group. Additionally, the GISTIC score was also observed to be higher in patients from the low CSI group compared to those in the high CSI group. Overall, CSI may provide valuable insights into the immune resistance mechanisms of high TMB tumors, underscoring its significance as a predictive biomarker for immune checkpoint inhibitors.

The tumor microenvironment has been established as a vital factor in the progression of various types of tumors. Tumor immune cell subpopulations vary among different tumor types and even among patients with the same pathological type (37). Through our selection process, we identified that SNRPE, correlated with the amplification of the long arm of chromosome 8, is linked to tumor stemness and encourages tumor cell proliferation. Previous research has demonstrated that SNRPE facilitates HCC tumorigenesis by regulating FGFR4 expression via alternative splicing mechanisms (38). In addition, we observed that patients with high SNRPE levels exhibited suppressed APC co-stimulation, Cytolytic activity, and HLA signatures, indicating that SNRPE can impede the activation and cytotoxic function of immune cells.

Tertiary lymphoid structures function as germinal centers for immune cells within the tumor microenvironment. In our study, we evaluated the expression levels of various interferons, interleukins, and their corresponding receptors that play roles in the formation of TLS (39). Our analysis demonstrated a considerable negative correlation between SNRPE expression and the levels of interleukins and interferons. Immune checkpoint inhibitors have emerged as a promising treatment strategy for advanced cancer. Higher levels of immune checkpoints facilitate tumor immune evasion and indicate a greater likelihood of response to these inhibitors. Additionally, we identified that several key immune checkpoints, such as TIM-3/HAVCR2, LAG3, PD-1/PDCD1, and PD-L2/PDCD1LG2, were significantly upregulated in the low SNRPE group. High expression of PD-L1 on tumor cells can bind to PD-L1 receptors on immune cells, initiating negative regulatory signals that impair T cell recognition of cancer cells, thereby allowing the tumor cells to evade the immune response (40). These results imply that patients with low SNRPE expression show an enhanced response to ICIs, likely due to the inhibition of TME components that support tumor progression, including the NOTCH1 signaling cascade, tumor necrosis factor (TGFB), and VEGF pathways. Furthermore, our findings confirmed that the overexpression of SNRPE notably boosted the proliferation and invasion abilities of ovarian cancer cells, indicating its potential as a therapeutic target for this type of cancer. In summary, these results suggest that SNRPE could affect the efficacy of immunotherapy by modifying the composition of the tumor microenvironment and influencing the recruitment of immune cells through its effects on chemokines and immune checkpoints.

While it is important to highlight the impressive accuracy of the CSI in predicting the success of immunotherapy, it is essential to acknowledge certain limitations in this study. The ability of the OV model to forecast outcomes of immunotherapy for ovarian cancer is based on projections generated by the submap algorithm, and the reliability of the CSI requires validation using real OV ovarian cancer immunotherapy groups.

## Conclusion

In summary, we have developed a reliable and consistent signature of CSCs by conducting an integrated analysis of CRISPR OV cell lines, large-scale OV tissues, and single-cell cohorts. This signature allows for the classification of OV patients and the prediction of outcomes for immunotherapy. Our research represents a groundbreaking exploration into the association between cancer stemness and immunotherapy in OV. It establishes a solid framework for understanding the importance of cancer stemness in immuno-oncology, clinical benefits, and practical implications. Based on our discoveries, this study enhances our comprehension of the link between cancer stemness and immunotherapy in OV, presenting new possibilities for treatment strategies.

## Data Availability

The original contributions presented in the study are included in the article/**Supplementary Material**. Further inquiries can be directed to the corresponding authors.

## Supplementary material

Supplementary Figure 1 Analysis of intrinsic somatic mutations and copy number variation patterns in ovarian cancer (OV) patients with differing CSI levels. **(A)** Waterfall plot depicting the mutation frequency of the top 15 genes in OV. **(B-E)** Box plots comparing tumor mutation burden, single nucleotide variant neoantigens, nonsilent mutation rate, and homologous recombination defects between low- and high-CSI groups. **(F)** GISTIC scores for low- and high-CSI groups in OV patients. (Wilcoxon test; * P < 0.05; ** P < 0.01; *** P < 0.001).

Supplementary Figure 2 SNRPE is highly expressed in EMT and proliferative tumor cells. **(A)** TSNE plot showing the composition of 5 main subtypes derived from OV malignant cells. **(B)** Dotplot showing the expression of SNRPE in malignant cell subtypes. **(C)** Dotplot showing the score of HALLMARK pathways in malignant cell subtypes. **(D)** There is a significant difference in cell-cell communication strength between SNRPE+ malignant and SNRPE- malignant cells with endothelial cells.

Supplementary Figure 3 Pan-cancer validation of the association between SNRPE and prognosis. **(A)** Univariate Cox regression analysis reveals a significant association between SNRPE and poor prognosis in various cancers.
