# Supplementary figures and images for: Multi-omics analysis and experiments uncover the function of cancer stemness in ovarian cancer and establish a machine learning-based model for predicting immunotherapy responses
Source: Front Immunol. 2024 Dec 11;15:1486652. doi: 10.3389/fimmu.2024.1486652 (PMC11670203; doi:10.3389/fimmu.2024.1486652)

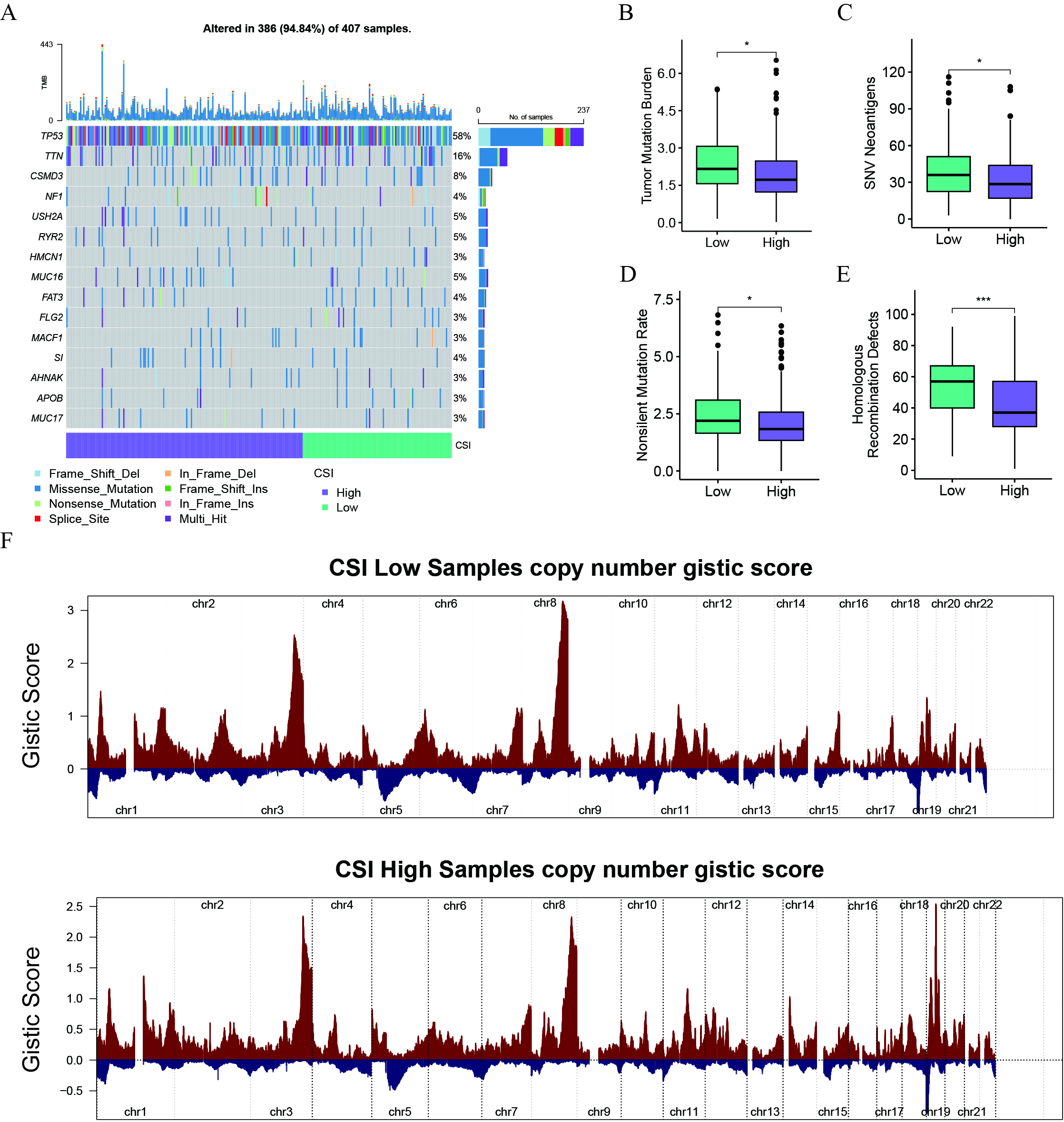

Supplement: Supplementary Figure 1 — Analysis of intrinsic somatic mutations and copy number variation patterns in ovarian cancer (OV) patients with differing CSI levels. (A) Waterfall plot depicting the mutation frequency of the top 15 genes in OV. (B-E) Box plots comparing tumor mutation burden, single nucleotide variant neoantigens, nonsilent mutation rate, and homologous recombination defects between low- and high-CSI groups. (F) GISTIC scores for low- and high-CSI groups in OV patients. (Wilcoxon test; * P < 0.05; ** P < 0.01; *** P < 0.001). [file Image1.tif]

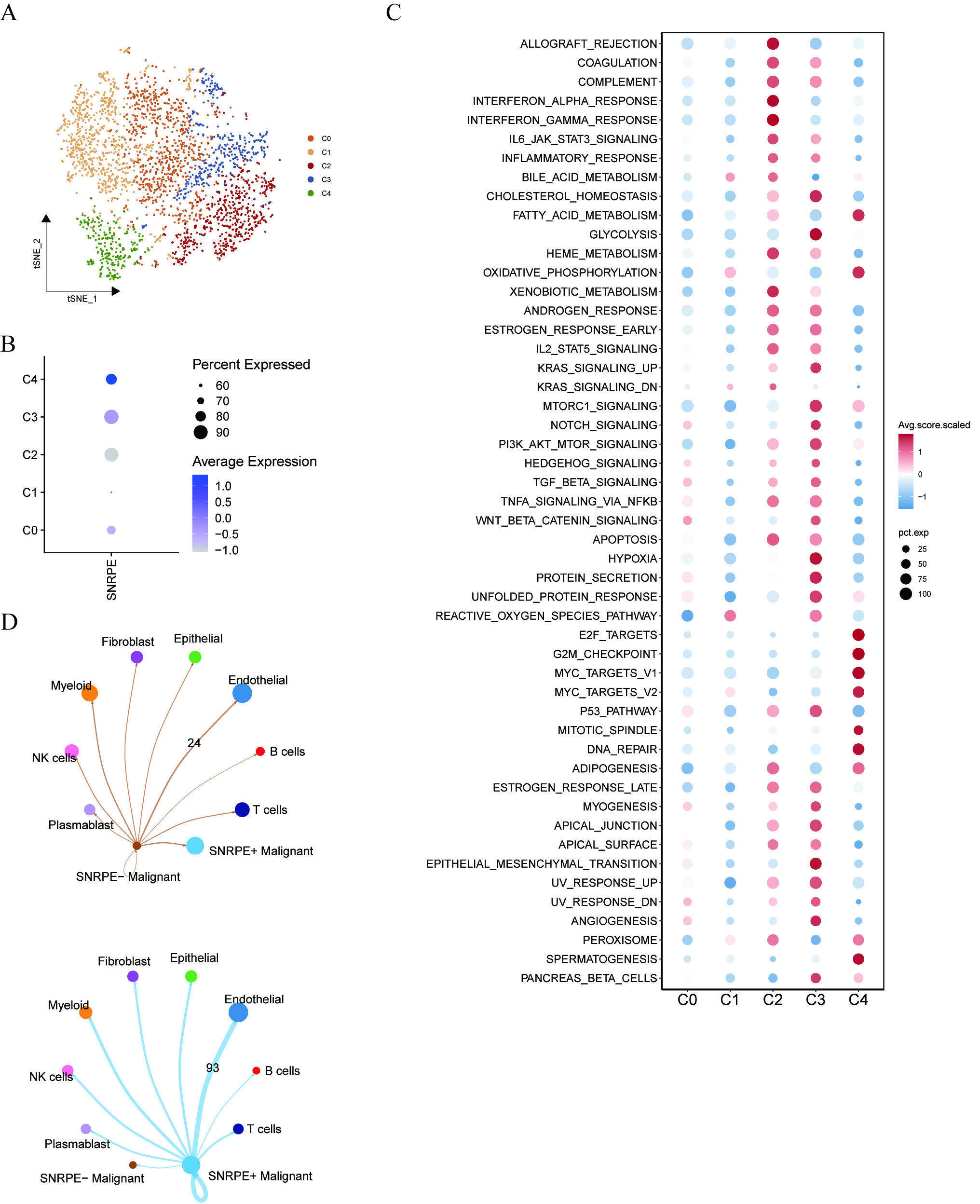

Supplement: Supplementary Figure 2 — SNRPE is highly expressed in EMT and proliferative tumor cells. (A) TSNE plot showing the composition of 5 main subtypes derived from OV malignant cells. (B) Dotplot showing the expression of SNRPE in malignant cell subtypes. (C) Dotplot showing the score of HALLMARK pathways in malignant cell subtypes. (D) There is a significant difference in cell-cell communication strength between SNRPE+ malignant and SNRPE- malignant cells with endothelial cells. [file Image2.tif]

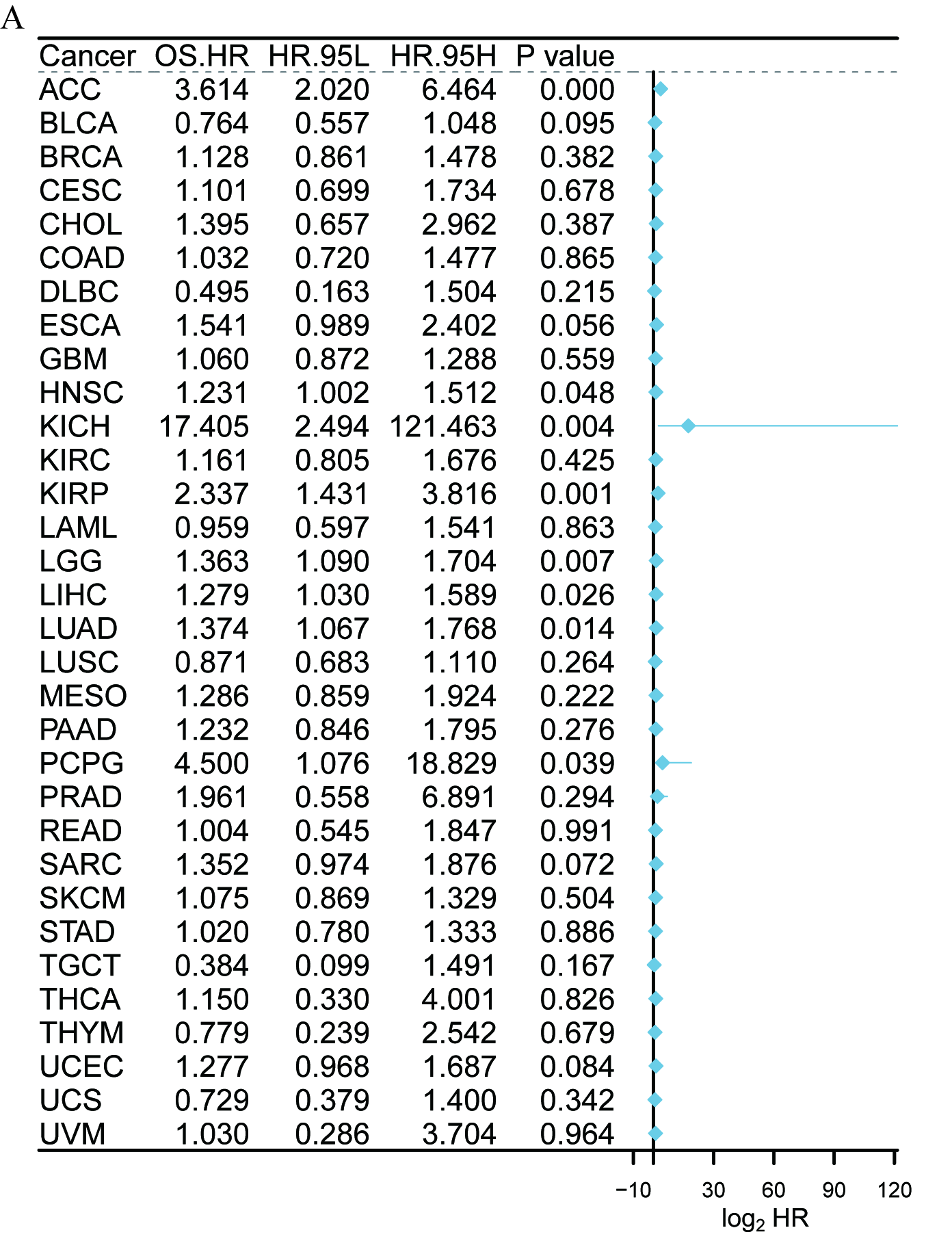

Supplement: Supplementary Figure 3 — Pan-cancer validation of the association between SNRPE and prognosis. (A) Univariate Cox regression analysis reveals a significant association between SNRPE and poor prognosis in various cancers. [file Image3.tif]
